# An Atypical Clinical Manifestation of a Hump-Nosed Pit Viper Envenomation

**DOI:** 10.1155/2019/4172395

**Published:** 2019-04-03

**Authors:** N. D. B. Ehelepola, C. N. Karunathilaka, G. L. H. S. Liyanage, W. A. C. B. Wickramaarachchi, J. R. P. U. Samarathunga, Wasantha P. Dissanayake

**Affiliations:** Teaching (General) Hospital Kandy, Kandy, Sri Lanka

## Abstract

Envenomations by hump-nosed pit vipers (HNVs) are frequent in Sri Lanka and in South India. Until recently, HNV was considered a moderately venomous snake. Here, we report a case of a previously healthy female developing all the known serious complications, plus some previously unreported complications following a HNV envenomation. She had muscarinic symptoms like profuse sweating and salivation within a couple of minutes and a seizure several minutes after envenomation. Her acute kidney injury (AKI) was swift onset and progressed to end-stage renal failure at three months. She had mild parotid swelling, crepitations in her lungs, and edema of the bitten leg. She had evidence of microangiopathic hemolytic anemia and hemolytic uremic syndrome as well. She developed local tissue necrosis, a non-ST-elevation myocardial infarction (non-STEMI), and anterior ischemic optic neuropathy (AION) following the envenomation. We believe the best explanation for her swift onset complication is intravascular injection of venom. We believe ischemia due to thrombotic microangiopathy has contributed to local tissue necrosis. Those ischemia and kidney failure have contributed to non-STEMI and AION. We illustrate the issue of the sluggish progress made by medicine in understanding the complications of envenomation by using HNV as an example.

## 1. Introduction

Pit vipers of the genus *Hypnale* are called hump-nosed pit vipers or simply hump-nosed vipers (HNVs) [1–4]. *Hypnale hypnale* (Merrem's hump-nosed pit viper), also known as *Trigonocephalus hypnale* in the past, is the commonest and most widely distributed species of the genus, and envenomation on people get reported from Sri Lanka and South India [1, 5]. *H. nepa* (*H. walli*) and *H. zara* are rare, and envenomation on people was reported [1]. Hitherto, only one specimen of *H. amal* has been identified, and there are no reports of envenomation [1]. The last three species are endemic to Sri Lanka [1]. According to past reports, as well as our experience, HNV envenomation usually results in local inflammation only or sometimes with hematological manifestations (coagulopathy) [2–4, 6–8]. Occasionally, acute kidney injury also ensues in addition [2–4, 6–8]. Until recently, HNVs were considered as a moderately venomous species by medical science; thus, HNV bites did not receive much attention [4, 6, 8]. Nonetheless, atypical life-threatening clinical manifestations and deaths after envenomation are increasingly being reported [1, 2, 4, 6–8]. Out of all the snakes with lethal venom in Sri Lanka, HNVs are responsible for hospital admissions with snakebites more than any other identified snakes in our practice as well as on a national level [2, 6–9]. The World Health Organization (WHO) now categorizes HNV as a class 1 (most significant) venomous snake [3]. No antisnake venom (ASV) is available against HNV venom [3, 6]. Here, we report an atypical manifestation of HNV envenomation and discuss related issues.

## 2. Case Presentation

A 47-year-old previously healthy Sinhala female's right foot was bitten by a snake near the back door of her home in the Kegalle district, Sri Lanka. Within seconds, she felt burning pain ascending along that limb, and there was heavy bleeding from the site of bite. Within a couple of minutes, she felt dizziness, nausea, and numbness of the whole body, had profuse sweating and frothy salivation, and was screaming in pain from the site of bite. On the way to the nearby hospital, she started to clench her jaw tightly and limbs became rigid; she was frothing and was not responding for about 5 minutes, indicating a generalized seizure. She arrived at the hospital within 30 minutes. The doctor at the outpatient department decided to administer ASV and directed the patient to an internal medicine ward for that. Physical examination findings at the ward were a pulse rate of 100/minute and blood pressure of 150/90 mmHg, and lungs were clear to auscultation bilaterally with an arterial oxygen saturation of 95% whilst breathing air with no neurological deficit. By this time, the killed snake was brought in and doctors identified it as a HNV; thus, antisnake venom (ASV) was not administered. Even though there was bleeding at the site of the bite even on admission to the hospital, her 20-minute whole blood clotting time, platelet count, prothrombin time and international normalized ratio, and activated partial thromboplastin time and liver function tests were all normal. Urine sample obtained via the catheter showed 50–55 red cells per high-power field, arterial blood gases indicated a compensated metabolic acidosis, and serum sodium and potassium levels were normal. Her urine output was <100 ml for the first 24 hours and serum creatinine rose from 80 *μ*mol/l to 277 *μ*mol/l. She was transferred to the Teaching Hospital, Kandy, on day 2 for further management.

On day 2, a bulla developed at the site of the bite, and there was an edema and warmth at the right foot. Complete (full) blood count demonstrated neutrophilic leucocytosis, and the CRP level of the following day was 261 mg/l. Intravenous antibiotics was started to cover the wound infection. Serum creatinine was 377 *μ*mol/l with oliguria on day 2. Serum sodium and potassium levels remained within the normal range from day 1–5. On the day 5, creatine kinase was 75.1 U/l. Regular hemodialysis every other day from day 2 to day 24 and fluid management were started. Oral sodium bicarbonate was started, and management of her acute kidney injury with collaboration of nephrology team continued.

**Figure 1:**
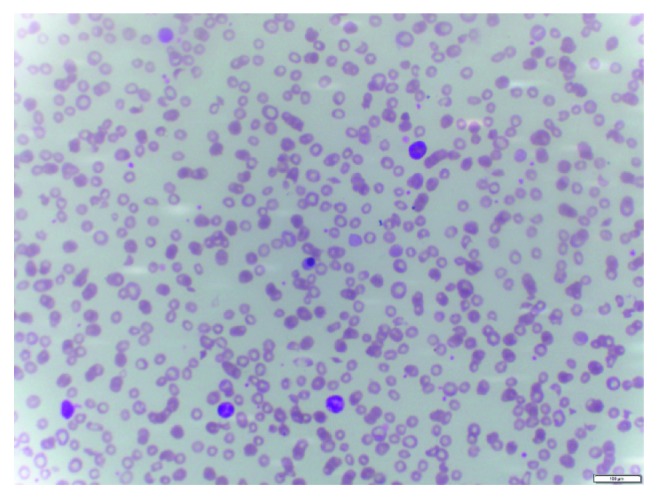
Blood film taken on day 11 depicting evidence of microangiopathic hemolytic anemia. A nucleated red blood cell is also seen.

On day 3, her blood pressure rose to 160/90 mmHg, and it was controlled by prazosin and nifedipine SR; however, it generally remained on or above 140/90 mmHg until her discharge. She developed bilateral lung crepitations on day 3 that remained for 7 days. She developed bilateral parotid swelling and edema of the right leg on day 3, and it lasted 3 days. Edema below her right knee persisted another 10 days. Her blood picture on day 2 did not show hemolysis and was suggestive of bacterial infection but blood picture on day 5 showed evidence of microangiopathic hemolytic anemia (MAHA), and same changes were there in a blood film taken on day 11, as depicted in Figure 1.

**Figure 2:**
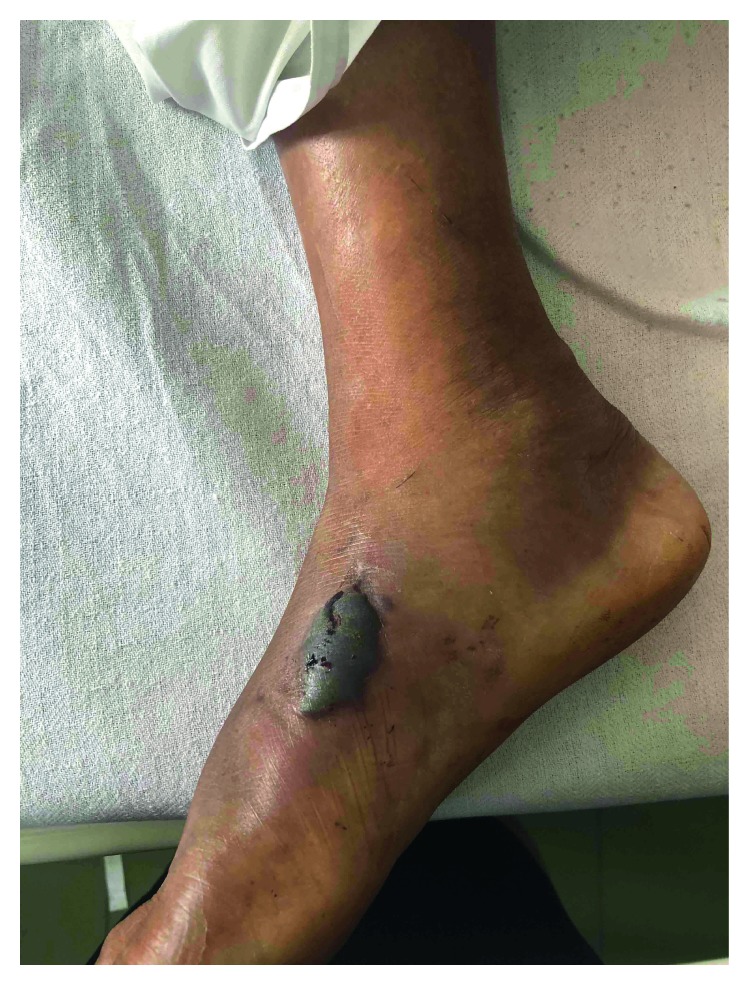
A bulla and tissue necrosis at the site of bite and edema of the right foot (day 6).

Her day 2 hemoglobin level of 10.8 g/dl dropped to 8.4 g/dl on day 5. On day 2, her platelet count was 104 × 10^9^/l and that dropped to nadir of 29 × 10^9^/l in day 6 and was <150 × 10^9^/l until day 20. A consultant in transfusion medicine has assessed her, and blood transfusion and plasmapheresis was performed on day 7. Another four cycles of plasmapheresis followed. Local edema at the site of the bite increased with necrosis (Figure 2); thus, wound debridement was done on day 7 and followed up by regular wound toilets.

We did an electroencephalogram (EEG) on this patient on the earliest available day (day 11) and that was normal. The 2D echocardiogram done on day 17 was also normal.

**Figure 3:**
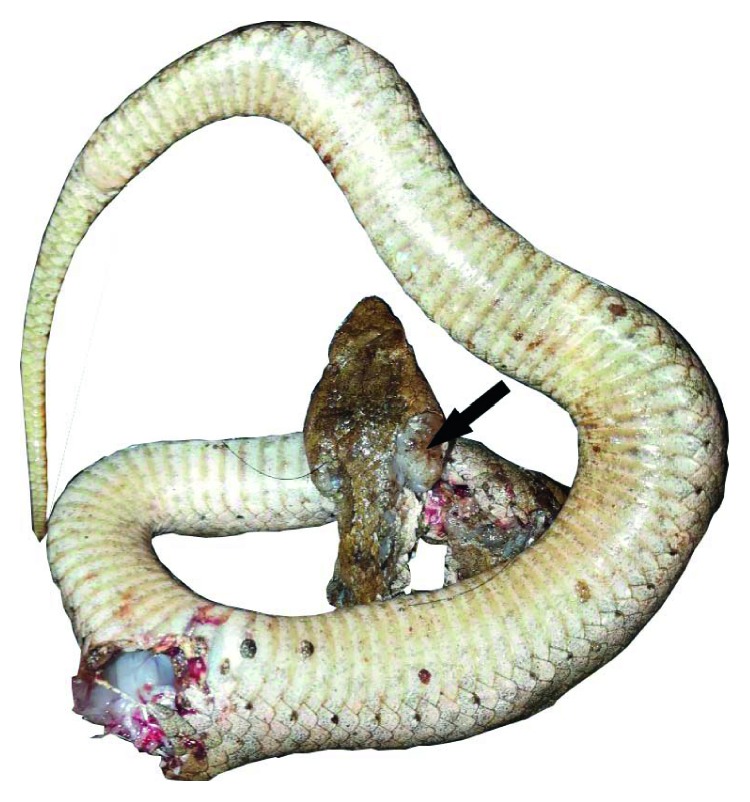
The offending snake. Large venom glands can be seen (pointed by an arrow). The pattern of scales in the dorsum of the head also contributed to positive identification of the species.

The offending snake's carcass was taken to the Peradeniya University, and an expert on HNV, Dr. Kalana Maduwage, has confirmed it as a *Hypnale hypnale*.  Figure 3 is a photo of the offending snake.

**Figure 4:**
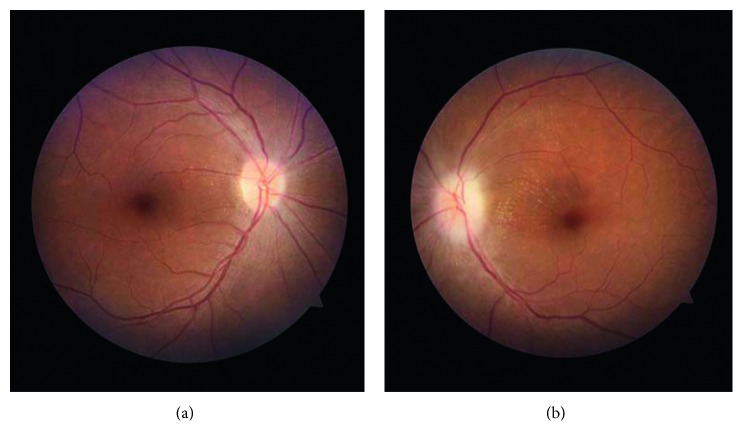
Photos of the fundi of this patient taken on day 77 after a month of steroid therapy. In the fundus of the right eye (a) and left eye (b), changes are mild after treatment. Bilateral optic disc edema more in the left and hemorrhage near left fundus are seen.

As her daily urine output improved to >1000 ml, she was discharged on day 30 and asked to come for a review in five days. She defaulted treatment and was on alternative medication. After developing progressive bilateral ankle edema and exertional dyspnea, she came back again on day 46, and hemodialysis and supportive therapy were restarted at the nephrology unit. On day 49, she had an anterolateral non-ST-elevation myocardial infarction (non-STEMI), and she was managed at the cardiology unit. She had progressive impaired vision of the left eye starting from a few days after the snakebite and could not count fingers held 30 cm in front of that eye on the 46th day. She was referred to the eye unit, there was bilateral optic disc edema more on the left, the patient was diagnosed of left anterior ischemic optic neuropathy (AION), and steroid therapy was started. Her erythrocyte sedimentation rate and contrast-enhanced computed tomography (CECT) brain done on day 53 were normal.  Figure 4 is a photograph of fundi of this patient.

She had two episodes of seizures on day 76, and we suspected a possible relationship to her envenomation. The opinion of the neurology team regarding three seizures was obtained. Repeated EEG and CECT brain were normal. Despite being on calcium carbonate 500 mg plus 0.25 *μ*g 1-alpha-hydroxycholecalciferol daily from day 46, her serum calcium level was low (1.8 mmol/l). Last two seizures were attributed to hypocalcemia due to chronic kidney disease following HNV envenomation, and daily calcium carbonate dose was increased to 500 mg thrice daily. After three months, she was diagnosed of end-stage renal disease by nephrology team and on hemodialysis once in four days and was searching for a kidney donor at six months.

## 3. Discussion

There were some unusual clinical manifestations in this patient that were previously not reported, after a bite by a HNV. In addition to severe pain at the site of the bite, which many other victims also have, this patient developed ascending burning pain, dizziness, nausea, sweating, numbness of the whole body, and excess salivation within couple of minutes. Although severe anxiety also may have contributed to those symptoms, we think systemic envenomation as the more likely cause. Aside from those, there was profuse and long lasting bleeding from the site of the bite which is uncommon and indicate the puncture of a blood vessel by a fang and intravascular injection of venom. This patient undergoing a generalized seizure after several minutes further supports this hypothesis. Usually, neurotoxic features are not seen after HNV bites [3, 6, 10]. The venom of vipers mainly consists of large molecules that usually do not cross the blood-brain barrier [11]. Nonetheless in a recently published series of 1543 hospitalized patients following HNV envenomation, three developed ophthalmoplegia and one fell into a coma [8]. There is a case report on a patient developing thrombotic thrombocytopenic purpura with a coma five days after envenomation by a HNV [12]. Seizures due to hyponatremia a day after a HNV bite were recently reported [13]. However, the serum sodium level of our patient went below 135 mmol/l (130 mmol/l) only once in day 4. We think the pathophysiology of the first seizure of this patient that occurred only several minutes after envenomation is different from those. The first seizure and symptoms of muscarinic effect symptoms are likely to be a result of direct neurotoxicity. Seizures were reported following the bites of the Malayan pit viper (*Calloselasma rhodostoma*), a phylogenetically closely related species to HNV [14]. EEG abnormalities devoid of any clinical neurological manifestations have been reported after HNV bites [3]. This patient's EEGs were normal. However, the first EEG was done on day 11. An *in vitro* study conducted using Sri Lankan HNV venom has shown mild neurotoxicity [15]. Venom from three *Hypnale* species has caused autonomic neurotoxicity with predominant muscarinic effects in mice in one study [16]. Interestingly, this patient also developed nausea and profuse sweating and salivation within a few minutes of the HNV bite, indicating autonomic neurotoxicity.

Patients coming in after being bitten by an unidentified snake are common in Sri Lanka [9, 10]. If such a patient comes in with signs of neurotoxicity, most doctors exclude the possibility of envenomation by a HNV [9, 10]. If a person bitten by an unidentified terrestrial snake (usually in the dark) in Sri Lanka and comes in with swift onset local edema and neurological manifestations, the norm for doctors is to think of an envenomation by a spectacled cobra (*Naja naja*) or a Western Russell's viper (*Daboia russelii*) and to administer ASV, as the first doctor who saw this patient also decided. This case indicates that in rare cases even HNV venom can result in those clinical features. Indian polyvalent ASV is the only ASV available in this country, and it does not cover HNV venom. Serious reactions to ASV is common, and it is costly [2, 17]. Thus, giving it to a patient like this one is useless and risky [2, 3]. However, polyvalent ASV covering venom of HNV and the two other snakes mentioned are being developed in Sri Lanka and that will be useful in similar circumstances [10].

Bringing the offending snake to the hospital for identification is a controversial issue. Considering their important role in the equilibrium in the environment, the concept of the killing of any animal is wrong (Buddhism is the faith of the majority of Sri Lankans), and the risk of handling the snake, killing the offending snake, and bringing it to hospitals are discouraged by many people including doctors at present. However, many people still do that. A few people take a photo from their smartphone and come. Nevertheless, a photo is sometimes inadequate for identification of the species as there can be Batesian mimicry, for example, nonvenomous wolf snakes (*Lycodon aulicus*) common in South Asia, including in Kandy, mimics highly venomous kraits (*Bungarus caeruleus* and *B. ceylonicus*), and a closer examination is necessary for the identification of snake species [3, 18]. In this particular case, bringing the dead snake to the hospital contributed to the correct management of this patient, and the reporting of unusual clinical features that we could confidently attributed to *H. hypnale* venom.

This patient had transient mild bilateral parotid swelling from days 3–6. She simultaneously developed bilateral lung crepitations and edema of the legs as well, indicating capillary leakage. Parotid swelling due to the capillary leak following Russell's viper bite is known and considered as a sign of poor prognosis [3]. This may be the first such report following the HNV bite but the swelling was mild, and we suspect that clinicians may be missing that because we are not accustomed to actively look for that sign in patients who come in following HNV bites.

In cases we have managed in the past and in reported cases of acute kidney injury (AKI) subsequent to HNV envenomation, AKI ensues on 3-4th day [12, 19], yet this patient had rapid onset AKI, and she passed only <100 ml urine during first 24 hours.

We think the best explanation for swift onset AKI and seizures is that venom from one fang was injected intravascularly and the venom of that bolus acted promptly on kidneys and brain, which receive high percentages of cardiac output. The first author, 17 years ago, had seen a young woman who died several minutes after bitten by a Western Russell's viper, and she had a fang mark puncturing a saphenous vein. There was severe local edema and necrosis indicating venom from the other fang deposited extravascularly. A study using rabbits has demonstrated that HNV venom injected intramuscularly (IM) has very low bioavailability compared to the intravenous route [20]. It is reasonable to presume subcutaneuos injection also may behave more like IM injections and that explains the slower onset and mild systemic symptoms seen in most cases of HNV envenomations. We think we can explain appearance of several complications, quickly, in her to the higher bioavailability of venom. This patient recovered the acute illness without the help of ASV, indicating the importance of adequate supportive care in saving the lives of such patients.

Thrombotic microangiopathy and resulting ischemic changes were histopathologically demonstrated in relation to small and medium arteries of the skin, kidneys, spleen, and myocardium following HNV envenomation [7]. We presume that as the likely mechanism resulting AION in the left eye, also contributed to non-STEMI in this patient and to necrosis near the site of the bite. Her kidney failure may also have contributed to the non-STEMI and AION [21]. However, we have no idea of the proportion of contribution from each of these factors and from any preexisting atheromatosis to her non-STEMI. There was no evidence of MAHA/hemolysis on day 2 but it was there on the day 5 blood picture, and there was no drop in her blood pressure. Considering the above, we believe her rapid onset AKI was probably initiated by toxins in the venom injected intravascularly but a few days later, thrombotic microangiopathy would also have aggravated the damage to kidneys [7].

Hemolytic uremic syndrome (HUS) following HNV envenomation has been reported [22]. Our patient had AKI, MAHA, and thrombocytopenia (day 2 to day 20), and she had a fever from day 2 to day 5 but we attributed her fever to infection at the site of the bite. We retrospectively think we could have diagnosed HUS following HNV envenomation in this patient [22].

The proteome of the *H*. *hypnale* venom in the descending order of relative abundance is phospholipase A_2_ (PLA_2_), snake venom metalloprotease (SVMP), snake venom serine protease (SVSP), L-amino acid oxidase (LAAO), and C-type lectin (CTL) [23]. The exact nature of their contribution to the symptoms of envenomation and their relative importance has yet to be established. PLA_2_ and SVMP may have contributed to her local tissue necrosis and edema, SVMPs may have resulted in capillary leakage that led to local edema, parotid swelling, and crepitations in the lungs, and LAAO is also known to contribute to tissue necrosis [23]. SVSP with the combination of others may have contributed to her thrombotic microangiopathy. PLA2, SVMP, and LAAO are large molecules that strongly bind to local tissues and result in tissue damage and that explain the low bioavailability of venom, thus the milder symptoms after intramuscular injection of venom [20, 23]. Therefore, there is a theoretical feasibility of reducing the severity of life-threatening systemic effects of HNV envenomation by delaying and reducing entry of those molecules to systemic circulation by a simple method like the prompt application of cold to the site of the bite for a certain period of time and by strict immobilization of the bitten limb.

We would like to illustrate the slow evolution of our understanding of snakebites in general and the effects of HNV envenomation in particular until recently, giving an example related to our hospital, because we believe that it is an important but neglected issue that deserves the attention of the medical community. Sometime after the last royal capital of Sri Lanka, Kandy, fell to the British (1815), Dr. John Davy, a British army surgeon, came to Kandy and contributed to establish the first allopathic hospital here and that evolved into the Teaching Hospital, Kandy, our institution [24]. The oldest published scientific study of pathophysiology of snake envenomation, according to the best of our knowledge, was conducted in Sri Lanka by Dr. John Davy and was published in Britain in 1821 [25]. Davy described local inflammation and necrosis following HNV bites on experimental animals [25] that has been common knowledge among Sri Lankans. *Hypnale* species collectively are locally known as “kunakatuwa” and by other names [2]. “Kunakatuwa” means “one who with a needle (fang) that gives rises to rotting (necrosis).” In the early 1990s, one author was a medical student and another was a medical officer at this hospital, and we remember the belief of our doctors and teachers of local medical schools was that HNV was still a moderately venomous snake and envenomation mostly only resulted in local effects; hence, HNV bites were not very important. Hematological and nephrotoxic effects were rarely reported [2] but gained wide acceptance only in the recent past. According to the latest (2014) systematic review on HNV bites and according to the latest edition of the WHO's publication “Guidelines for the management of snake-bites” as well as according to the 2018 edition of the standard handbook published by the Sri Lankan Health Ministry and used by most doctors treating snakebites in this country, there is no evidence of direct neurological toxicity following HNV envenomation [3, 4, 6]. Our case and some past reports indicate HNV envenoming can result in clinical neurotoxicity in people [8]. We think that the generation of more scientific information on the topic and updating the knowledge of doctors accordingly is essential. Nevertheless, many local doctors treating snakebites find it difficult to find the time and resources to publish unusual findings. Nonetheless, we have noticed a silver lining: the proliferation of both portable electronic devices and electronic publications in the present decade and that has enabled an average Sri Lankan doctor to easily, freely, and quickly access to the latest information on medical topics, including HNV bites, and to publish interesting cases. We think that is an important factor, among others, that contributed to the recent rise of publications on snakebites. These helped to update the medical community, including ourselves, on a wider spectrum of clinical problems following HNV envenomation.

## Data Availability

All the information supporting our conclusions and relevant references is included in the manuscript. There are no data sheets related to this case report.

## Abbreviations

HNV: Hump-nosed pit vipers (hump-nosed vipers)
AKI: Acute kidney injury
Non-STEMI: Non-ST-elevation myocardial infarction
AION: Anterior ischemic optic neuropathy
WHO: World Health Organization
ASV: Antisnake venom
MAHA: Microangiopathic hemolytic anemia
CRP: C-reactive protein
EEG: Electroencephalogram
CECT: Contrast-enhanced computed tomography
IM: Intramuscular
HUS: Hemolytic uremic syndrome.
